# Acetate correlates with disability and immune response in multiple sclerosis

**DOI:** 10.7717/peerj.10220

**Published:** 2020-11-16

**Authors:** Silvia Pérez-Pérez, María Inmaculada Domínguez-Mozo, Aitana Alonso-Gómez, Silvia Medina, Noelia Villarrubia, Jose Ignacio Fernández-Velasco, María Ángel García-Martínez, Estefanía García-Calvo, Héctor Estévez, Lucienne Costa-Frossard, Jose C. Alvarez-Cermeño, Jose L. Luque-Garcia, Rafael Arroyo, Luisa M. Villar, Roberto Alvarez-Lafuente

**Affiliations:** 1 Grupo de Investigación de Factores Ambientales en Enfermedades Degenerativas, Instituto de Investigación Sanitaria Hospital Clínico San Carlos (IdISSC) / Red Española de Esclerosis Múltiple (REEM), Madrid, Spain; 2 Servicio de Inmunología, Hospital Universitario Ramón y Cajal. Instituto Ramón y Cajal de Investigación Sanitaria (IRYCIS) / Red Española de Esclerosis Múltiple (REEM), Madrid, Spain; 3 Department of Analytical Chemistry, Faculty of Chemical Sciences, Universidad Complutense de Madrid, Madrid, Spain; 4 Servicio de Neurología, Hospital Universitario Ramón y Cajal / Red Española de Esclerosis Múltiple (REEM), Madrid, Spain; 5 Department of Neurology, Hospital Universitario Quironsalud Madrid / Red Española de Esclerosis Múltiple (REEM), Madrid, Spain

**Keywords:** Multiple sclerosis, Short chain fatty acids, Acetate, Plasma, EDSS

## Abstract

**Background:**

Gut microbiota has been related to multiple sclerosis (MS) etiopathogenesis. Short-chain fatty acids (SCFA) are compounds derived from microbial metabolism that have a role in gut-brain axis.

**Objectives:**

To analyse SCFA levels in plasma of MS patients and healthy donors (HD), and the possible link between these levels and both clinical data and immune cell populations.

**Methods:**

Ninety-five MS patients and 54 HD were recruited. Patients were selected according to their score in the Expanded Disability Status Scale (EDSS) (49 EDSS ≤ 1.5, 46 EDSS ≥ 5.0). SCFA were studied in plasma samples by liquid chromatography-mass spectrometry. Peripheral blood mononuclear cells were studied by flow cytometry. Gender, age, treatments, EDSS and Multiple Sclerosis Severity Score (MSSS) were evaluated at the recruitment.

**Results:**

Plasma acetate levels were higher in patients than in HD (*p* = 0.003). Patients with EDSS ≥ 5.0 had higher acetate levels than those with EDSS≤ 1.5 (*p* = 0.029), and HD (*p* = 2.97e–4). Acetate levels correlated with EDSS (*r* = 0.387; *p* = 1.08e–4) and MSSS (*r* = 0.265; *p* = 0.011). In untreated MS patients, acetate levels correlated inversely with CD4+ naïve T cells (*r* =  − 0.550, *p* = 0.001) and directly with CD8+ IL-17+ cells (*r* = 0.557; *p* = 0.001).

**Conclusions:**

Plasma acetate levels are higher in MS patients than in HD. In MS there exists a correlation between plasma acetate levels, EDSS and increased IL-17+ T cells. Future studies will elucidate the role of SCFA in the disease.

## Introduction

Multiple sclerosis (MS) is a chronic autoimmune disease of the central nervous system (CNS) causing inflammation, demyelination, and neurodegeneration (Compston & Coles, 2008). Although its etiology remains unclear, several environmental and genetic risk factors seem to be involved in its development (Belbasis et al., 2015). Recently, gut microbiota has been added to this list.

The gut microbiota is considered as the pool of bacteria, archaea, fungi, eukaryotes and viruses that reside in mucosal surfaces of the intestine. The gut bacteria mainly include six major phyla: Actinomycetes, Bacteroidetes, Firmicutes, Fusobacteria, Proteobacteria and Verrucomicrobia (Eckburg et al., 2005).

After many years of common development, the gut bacteria have reached a symbiotic state with the human body. Gut microbiota may affect the CNS and participate in the regulation of nervous system function. The CNS also plays an important role in regulating the gut function and homeostasis. This complex relationship may be included in the new concept: the microbiota gut-brain axis (Zhu et al., 2017).

When the mutualistic relationship between the host and microbiota is disrupted, the gut microbiota can cause or contribute to different pathologies, such as MS (Littman & Pamer, 2011). During the last years, several groups studied the dysbiosis of the gut microbiota in MS. Two main approaches have been addressed. The first one was based in the experimental autoimmune encephalomyelitis (EAE) model. The alteration of the gut microbiota diminished the EAE frequency and mitigated the severity of the disease (Ochoa-Repáraz et al., 2011).

In the second approach, the microbiota of MS patients was studied by several groups that showed the presence of gut microbial dysbiosis in MS compared with healthy controls (Lee et al., 2011).

Some microorganism-derived molecules as short-chain fatty acids (SCFA) can have a role in the microbiota gut-brain axis (Zhu et al., 2017). They are produced from the dietary fibers fermented by gut microorganisms in the large intestine. The most abundant are butyrate, propionate, and acetate (95% of SCFA) (Wong et al., 2006). Bacteria of the Bacteroidetes phylum produce high levels of acetate and propionate, whereas bacteria of the Firmicutes phylum produce high amounts of butyrate (Macfarlane & Macfarlane, 2003). Then, it is logical to think that the levels of SCFAs could be modified in MS patients compared with healthy population, considering that both phylum Bacteroidetes and Firmicutes are altered in these patients and have important immunoregulatory functions (Tremlett et al., 2016).

Previous metabolomic studies performed in MS patients showed higher levels of acetate in the serum of MS patients in comparison with healthy controls (Moussallieh et al., 2014). Furthermore, a previous pilot study performed by our group showed significantly higher levels of acetate among MS patients with higher scores in the Expanded Disability Status Scale (EDSS) in comparison to those MS patients with lower EDSS scores (results are shown in the Table S1; original data are available in a Supplemental File). Based on these previous results, we designed the current study to analyze the possible differences in the levels of circulating SCFA in two groups of MS patients (those without disability, EDSS ≤ 1.5, and those with severe disability, EDSS ≥ 4.5) and in healthy donors (HD). To study the immunoregulatory functions of the SCFA, we also analyzed their possible correlations with blood immune cell subsets and if this function was the same or not in MS patients and healthy controls. Since immunomodulatory and immunosuppressive treatments modify cell subsets, around 50% of MS patients recruited in each group were without treatment to perform cell studies by flow-cytometry. The last aim of the study was to analyze possible correlations between circulating SCFA levels and different clinical and demographic characteristics.

## Materials & Methods

### Subjects

A total of 95 patients with clinically definite RR-MS and 54 HD from “Hospital Universitario Ramón y Cajal” and “Hospital Clínico San Carlos” were included in the study. MS patients were recruited based on their EDSS score: 49 MS patients patients had EDSS between 0 and 1.5 and 46 between 5.0 and 7.5 (Table 1).

**Table 1:** Demographical characteristics of the patients included in the study.

	**MS vs HD**			**MS groups**		
	**MS**	**HD**	***p***	**EDSS ≤ 1.5**	**EDSS ≥ 5**	***p***
**N**	95	54	–	49	46	–
**GENDER (%)**						
MALES	31.6	38.9	0.371	28.6	34.8	0.661
FEMALES	68.4	61.1		71.4	65.2	
**AGE (YEARS, MEDIAN (P25-P75))**	43.0 (39.0–48.0)	43.0 (37.3–48.0)	0.609	40.0 (36.0–44.0)	47.5 (42.3–52.5)	0.001
**DISEASE DURATION (MONTHS, MEDIAN (P25-P75))**	118.0 (69.0–191.5)	–	–	81.0 (36.0–123.0)	183.0 (108.1–256.5)	0.001
**CURRENT TREATMENT (%)**						
IMMUNOMODULATOR (INTERFERON BETA; GLATIRAMER ACETATE)	36.2	–	–	45.8	26.1	
IMMUNOSUPPRESSANT (NATALIZUMAB; FINGOLIMOD)	16.0	–		4.2	28.3		0.004
WITHOUT TREATMENT	47.9	–		50.0	45.7	
**TREATMENT DURATION (MONTHS, MED (P25-P75))**	29.5 (3.8–76.5)	–	–	53.0 (3.0–75.0)	17.0 (6.0–77.0)	0.755
**EDSS (MEDIAN (P25-P75))**	1.5 (1.0–6.0)	–	–	1.0 (0.0–1.5)	6.0 (5.6–6.5)	1.00e−13
**MSSS (MEDIAN (P25-P75))**	4.3 (0.8–6.6)	–	–	0.8 (0.5–1.8)	6.6 (5.3–7.9)	1.00e−13

### Ethics Statement

The study was approved by the Ethics Committee (Comité Ético de Investigación Clínica del Hospital Clínico San Carlos - 17/270-E_BS) and all patients signed an informed consent.

### Collection of samples

All samples were collected in the morning, between 8 and 11 AM. Two cell preparation tubes with sodium citrate (CPTTM, BD Vacutainer^®^) were collected for cell and plasma isolation. Peripheral blood mononuclear cells (PBMCs) were isolated using density gradient centrifugation (920 g, 30 min); they were cryopreserved in fetal bovine serum (FBS) with DMSO (10%) and stored in liquid nitrogen (−196 °C). After centrifugation, plasma was aliquoted and stored at −80 °C.

### Liquid chromatography-Mass spectrometry (LC-MS/MS) chemicals

Acetic, butyric and propionic acids standards and the compounds used for derivatization: 3-Nitrophenylhydrazine hydrochloride (3-NPH), N-(3-dimethylaminopropyl)-N’-ethylcarbodiimide hydrochloride (EDC), formic acid, and pyridine were purchased from Sigma-Aldrich. LC-MS water and acetonitrile were provided by Fischer Scientific.

### LC-MS/MS standard preparation

The stock standard solution containing 5 g L-1 of acetic, propionic and butyric acid was prepared in acetonitrile:water1:1(v/v). Derivatization was carried out by adding to 40 µL of the standard mixture 20 µL of 200 mM 3-NPH and 20 µL of 120 mM EDC in 6% pyridine. The mixture was incubated during 30 min at 40 ° C and further diluted up to two mL in 10% acetonitrile. Before analysis, samples were diluted in a 1:1 ratio using ACN:H2O 1:1 (v/v) and filtered with 0.22 µm PTFE filters.

### LC-MS/MS plasma sample preparation

Proteins were precipitated using an equal volume of ACN:H2O 1:1 (v/v). After 10 min of centrifugation at 500 g and 4  ° C. Derivatization was carried out as previously described for the standard mixture.

### LC-MS/MS analysis

A 8,030 Shimadzu triple-quadrupole mass spectrometer equipped with an ESI ionization source and operating in negative mode was used for determination of the selected metabolites. The chromatographic separation was performed using a Phenomenex Gemini C18 (5 µm, 150 × 2 mm) column, using 0.01% formic acid in water (solvent A) and 0.01% formic acid in acetonitrile (solvent B). The gradient program was as follows: 20% phase B was used as initial mobile phase for 2 min. Two linear gradients were established in order to reach first, a 40% phase B composition in 5 min and then, a final 100% phase B composition in 0.5 min. 100% phase B was maintained for 0.5 min and finally, 2 min were necessary for reestablishing the initial conditions. Samples were delivered at a flow rate of 0.6 mL min-1. Column temperature was set to 40 °C and autosampler temperature was set to 4 °C. A sample volume of 10 µL was analyzed in all cases.

The multiple reaction monitoring (MRM) transitions (Table 2) were selected by direct infusion into the mass spectrometer of a mixture of the 3 derivative compounds at 5 mg L^−1^. Mass range for full-mass Q1 scans was set between 100-1000 m/z using a 0.309 s scan time. Optimization of the collision energy for the precursor ions was carried out by ramping from 10 to 120 V in the collision cell. Fragment ions were detected in Q3 obtaining Q1/Q3 pairs for each analyte. Nitrogen gas was employed as nebulizing (1.5 L min^−1^), and drying gas (15 L min ^−1^), while argon served as collision gas (17 kPa). ESI capillary voltage was set to 4.5 kV and the DL temperature to 250  ° C. Data were obtained and processed with LabSolution software. One of the MRM transitions was used for quantitation and the other one acted as qualifier for verifying the compound identity. Table 2 shows all MRM transitions for the three analytes.

**Table 2:** MRM transitions of 3-NPH derivatized acids.

Compound	Q1 m/z	Q3 m/z	Collision energy (V)
Acetate	194.0	152.1	18
Propionate	208.2	137.05	20
Butyrate	221.1	137.00	20

### Monoclonal antibodies

For the study of the cell populations, the following monoclonal antibodies were used: Granulocyte/macrophage-colony stimulating factor (GM-CSF)-PE, CD197-PE (CCR7-PE), CD24-PE, Interferon (IFN)-gamma-FITC, CD14-FITC, CD8-FITC, CD27-FITC, CD8-APC-H7, CD4-APC-H7, CD56-APC, CD45RO-APC, CD3-BV421, CD127-BV421, CD45-V500 TNF-alpha-PerCP-Cy5.5, CD38-PE-Cy5.5, CD19-PE-Cy7, CD25-PE-Cy7, CD3-PerCP, (BD Biosciences, San Diego, CA, USA) and IL-17-APC (R&D Systems, Minneapolis, MN, USA).

### In vitro stimulation and labelling of antigens

Briefly, 10^6^ PMBCs were labelled with monoclonal antibodies for membrane antigen staining; then, PBMCs were washed with PBS and finally analyzed in a flow cytometer (FACSCanto II. BD Biosciences). Intracellular cytokine detection was performed stimulating 10^6^ PMBCs with 50 ng/mL Phorbol 12-myristate 13-acetate (PMA) and 750 ng/mL Ionomycin (Sigma-Aldrich, St. Louis, MO), in presence of 2 µg/mL Brefeldin A and 2.1 µM Monensin (BD Biosciences), during 4 h. After the cells were stained with monoclonal antibodies recognizing the surface antigens, PBMCs were fixed and permeabilized with Cytofix/Cytoperm Kit (BD Biosciences), and stained intracellularly with monoclonal antibodies recognizing IL-17, IFN-gamma, TNF-alpha and GM-CSF cytokines. Cells were finally analyzed in a flow cytometer (FACSCanto II. BD Biosciences).

### Flow cytometry

A gate including lymphocytes and monocytes and excluding debris and apoptotic cells was first established (Fig. S1). CD4+ and CD8+ T cells were classified as: central memory (CM) (CCR7+ CD45RO+); naïve (CCR7+ CD45RO-); terminally differentiated (TD) (CCR7- CD45RO-); effector memory (EM) (CCR7- CD45RO+). Regulatory CD4 T cells (Treg) were defined as CD3+ CD4+ CD25hi CD127low. B cells were classified as: plasmablasts (CD19+ CD27hi CD38hi), memory (CD19+ CD27dim CD38dim) and regulatory B cells (Breg) (CD19+ CD27- CD24hi CD38hi); natural killer (NK) cells (CD56dim CD3-), natural killer T (NKT) cells (CD56dim CD3+), and CD56bright NK cells (CD3- CD56bright) were also studied. Data analysis was performed using FACSDiva Software V.8.0 (BD Biosciences).

### Statistical analysis

Continuous variables were expressed as median (25th, 75th percentile) and categorical variables as percentages. We described the association between different demographical and clinical variables to the levels of acetate, propionate and butyrate in plasma. For this purpose, the continuous variables between groups were compared using the Mann–Whitney U-test and the Spearman’s rank correlation coefficient was used when studying the relationship between two continuous variables, included the percentages of the different immune populations. In case of multiple comparisons, the Bonferroni correction was applied. Subjects with missing data were omitted from the corresponding analyses. *P*-values ≤ 0.05 were referred to as statistically significant in the text. All analyses were performed using SPSS for Windows (Ver. 15.0) software (SPSS Inc, Chicago, IL, USA).

**Figure 1:**
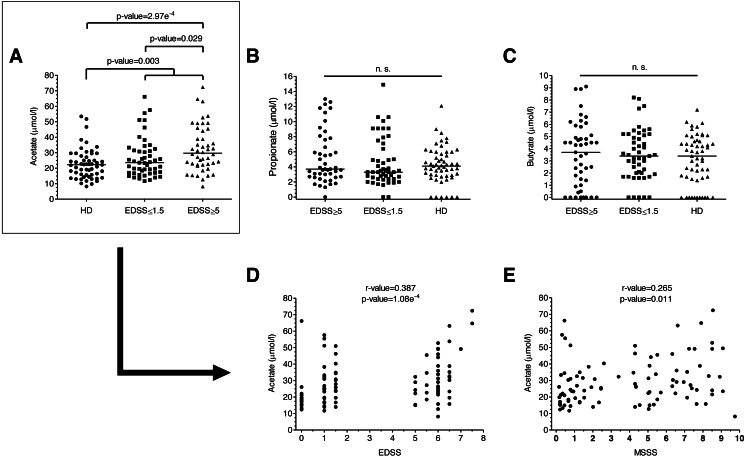
SCFAs levels of MS patients and HD. (A–C) SCFAs levels of MS patients and HD. Plasma acetate levels were significantly higher in MS patients than in HD (*p* = 0.003). Specifically, they were higher in patients with more disability (EDSS ≥ 5) than both in patients with EDSS ≤ 1.5 (0.029) and HD (2.97e−4). (D–E) Disability scores (EDSS and MSSS) correlated positively with plasma acetate levels. (Mann–Whitney *U*-test has been performed for A–C, and Spearman’s rank correlation coefficient for D–E).

## Results

### Short-chain fatty acids in MS patients and healthy controls

We analyzed plasma acetate, propionate and butyrate levels in all the samples included in the study (Figs. 1A–1C); original data are available in supplementary files. There were no significant differences in levels in terms of gender, age or treatments. Regarding the number of relapses 6 months before and 6 months after the sample collection, we did not find any correlation with anyone of the SCFA. Plasma acetate (but not propionate or butyrate) levels were significantly higher in MS patients (Fig. 1A). Moreover, those with EDSS ≥ 5 showed higher acetate levels than those with EDSS ≤ 1.5, existing a positive correlation between acetate levels and EDSS (Fig. 1D). MSSS (Multiple Sclerosis Severity Score), another disability score used in MS, also correlated with acetate levels (Fig. 1E).

We also analyzed the possible relationship among SCFA. All of them were correlated in MS patients; by contrast, in HD only acetate and propionate were correlated (Table 3).

**Table 3:** Correlation among SCFA according to the Spearmans rank correlation coefficient (*r* value).

		MS	HD
Acetate–Propionate	*r*-value *p*-value	0.438 9.93e^−6^	0.339 0.011
Acetate–Butyrate	*r*-value *p*-value	0.308 0.002	−0.017 0.900
Propionate–Butyrate	*r*-value *p*-value	0.714 6.95e^−16^	0.237 0.081

### Immune cell subsets

We next explored the possible correlation between SCFA plasma levels and immune cells. Since it has been described that immunomodulatory and immunosuppressive treatments modify cell subsets, we only analyzed PBMCs by flow cytometry in non-treated MS patients and HD. Results are shown in Tables 4 and 5. In MS patients, we found an inverse correlation between acetate levels and naïve CD4+ T lymphocytes and a direct correlation between acetate levels and CD8+ T cells producing IL-17 (see Fig. S2). We also found a direct correlation between propionate and butyrate levels with CD8+ T cells producing IL-17. Finally, propionate were inverse correlated with memory B lymphocytes on HD.

**Table 4:** Correlations between plasma SCFA levels and immune cells populations for MS patients (Spearmans rank correlation coefficient).

	Acetate *r*-value (*p*-value)	Propionate *r*-value (*p*-value)	Butyrate *r*-value (*p*-value)
CD4+ T	ns	ns	ns
CD4+ N	**−0.550 (0.001)^a^**	−0.425 (0.019)	ns
CD4+ CM	ns	ns	ns
CD4+ EM	ns	ns	ns
CD4+ TD	ns	ns	ns
CD4+ IFN-*γ*+	ns	ns	ns
CD4+ TNF-*α*+	ns	ns	ns
CD4+ IL-17+	0.441 (0.015)	ns	0.419 (0.021)
CD4+ GM-CSF+	ns	ns	0.513 (0.010)
CD8+ T	ns	0.407 (0.017)	ns
CD8+ N	ns	ns	ns
CD8+ CM	ns	ns	ns
CD8+ EM	ns	ns	0.427 (0.021)
CD8+ TD	ns	ns	ns
CD8+ IFN-*γ*+	ns	ns	ns
CD8+ TNF-*α*+	ns	ns	ns
CD8+ IL-17+	**0.557 (0.001)^a^**	**0.573 (0.001)^a^**	**0.560 (0.001)^a^**
CD8+ GM-CSF+	ns	ns	0.464 (0.022)
Treg	ns	ns	ns
B cells	ns	ns	ns
Bmem	−0.370 (0.044)	ns	ns
PB	ns	ns	ns
Breg	ns	ns	ns
CD19+ TNF- *α*+	−0.522 (0.004)	−0.501 (0.007)	ns
CD19+ GM-CSF+	0.418 (0.047)	ns	0.430 (0.041)
NK	ns	ns	ns
NKT	ns	ns	ns
CD56+ bright (NKreg)	0.498 (0.010)	0.467 (0.016)	0.397 (0.044)
Monocytes	ns	ns	ns

**Notes.**

a Values in bold are statistically significant results after Bonferroni correction (*p*-value ≤ 0.002).

ns not significant N naïve CM central memory EM effector memory TD totally differentiated IFN-*γ* interferon-gamma TNF-*α* tumor necrosis factor-alpha IL-17 interleukin 17 GM-CSF granulocyte/macrophage colony stimulating factor Treg regulatory T cells Bmem memory B cells PB plasmablasts Breg CD27-regulatory B cells NK natural killer NKT natural killer T cells NKreg NK regulatory cells

**Table 5:** Correlations between plasma SCFA levels and immune cells populations for HD (Spearman’s rank correlation coefficient).

	Acetate *r*-value (*p*-value)	Propionate *r*-value (*p*-value)	Butyrate *r*-value (*p*-value)
CD4+ T	ns	ns	ns
CD4+ N	ns	ns	ns
CD4+ CM	ns	ns	ns
CD4+ EM	ns	0.424 (0.031)	ns
CD4+ TD	0.397 (0.045)	ns	ns
CD4+ IFN-*γ*+	ns	ns	ns
CD4+ TNF-*α*+	ns	ns	ns
CD4+ IL-17+	ns	0.390 (0.049)	ns
CD4+ GM-CSF+	0.479 (0.013)	ns	ns
CD8+ T	ns	ns	0.432 (0.027)
CD8+ N	ns	ns	ns
CD8+ CM	ns	ns	ns
CD8+ EM	ns	ns	ns
CD8+ TD	ns	0.481 (0.013)	ns
CD8+ IFN-*γ*+	ns	ns	ns
CD8+ TNF-*α*+	ns	ns	ns
CD8+ IL-17+	ns	ns	ns
CD8+ GM-CSF+	0.489 (0.011)	ns	ns
Treg	ns	ns	ns
B cells	ns	−0.608 (0.013)	ns
Bmem	ns	**0.638 (0.001)^a^**	ns
PB	ns	ns	ns
Breg	ns	ns	ns
CD19+ TNF- *α*+	ns	ns	ns
CD19+ GM-CSF+	ns	−0.550 (0.004)	ns
NK	−0.332 (0.041)	ns	ns
NKT	ns	ns	ns
CD56+ bright (NKreg)	ns	ns	ns
Monocytes	ns	0.562 (0.003)	ns

**Notes.**

a Values in bold are statistically significant results after Bonferroni correction (*p*-value ≤ 0.002).

ns not significant N naïve CM central memory EM effector memory TD totally differentiated IFN- *γ* interferon-gamma TNF- *α* tumor necrosis factor-alpha IL-17 interleukin 17 GM-CSF granulocyte/macrophage colony stimulating factor Treg regulatory T cells Bmem memory B cells PB plasmablasts Breg CD27-regulatory B cells NK natural killer NKT natural killer T cells NKreg NK regulatory cells

## Discussion

The SCFA are bacterial metabolites mainly produced during the colonic fermentation of undigested carbohydrates. There are evidences for their regulatory role in host metabolism: they are ligands for free fatty acid receptor 2 and 3 (FFAR 2/3), found on a wide range of cell types, (Morrison & Preston, 2016), and have been recognized as mediators of immune responses (Asarat et al., 2016).

Acetate production pathways are widely distributed among bacterial groups whereas pathways for propionate and butyrate production appear more highly conserved and substrate specific (Morrison & Preston, 2016). Changes in the circulating levels of these metabolites could reflect variations in the bacteria composition of the gut microbiota. Dysbiosis has been observed in different pathologies, including MS (Jangi et al., 2016).

Furthermore, it has been suggested that the microbiota ability to produce SCFA, rather than the microbiota “composition”, is essential to generate the benefits linked to fibres (Mithieux, 2018). The plasma SCFA levels of MS patients have not been fully addressed yet. We explored them in our cohort and did not find significant differences in the levels of propionate and butyrate between MS patients and controls. However, MS patients showed significantly higher plasma acetate levels than HD (*p* = 0.003). These levels also correlated with the degree of disability, being significantly increased in patients with high disability than in patients with low or no disability (*p* = 0.029) and in HD (2.97e−4). Moreover, we showed a correlation between plasma acetate levels and the degree of disability, both with the EDSS (*r*-value = 0.387, *p*-value = 1.08e−4) and the MSSS (*r*-value = 0.265, *p*-value = 0.011). These results do not agree with a recent publication (Duscha et al., 2020). The authors find lower levels of propionate in MS patients than in HD, but they do not find any significant difference for acetate or butyrate. A possible explanation for this discordant results could be found in the patients selection. In this study, based on previous results of our group, we selected two groups of MS patients: those without disability, EDSS ≤1.5, and those with severe disability, EDSS ≥ 4.5, while in the study of Duscha et al. patients were not selected by their EDSS. As we have previously mentioned, a statistical significant difference was found between HD and patients with EDSS ≥ 4.5 (2.97e−4), but we did not find any statistical significant difference when we compared acetate levels between HD and patients with lower EDSS.

To study the possible mechanisms by which SCFA could contribute to disability we studied different PBMC subsets. In spite of not having found differences in SCFA levels among patients differently treated, we only performed flow cytometry analysis in samples from non-treated patients, because it has been recently described that some treatments, such as dimethyl fumarate and glatiramer acetate, could affect microbiota composition (Sand et al., 2019). Increases in plasma acetate levels correlated with a decrease of CD4+ naïve cells and with higher CD8+ T cells producing IL-17. By contrast, the only association that remained significant in control group was an inverse correlation between plasma propionate levels and memory B cells. CD8+ T cells are the most frequent immune cells present in MS lesions, and they have been involved in inflammation and axonal damage (Bar-Or et al., 2010; Salou et al., 2015). In addition it has been postulated that CD8+ IL-17+ T cells play an important role in MS pathology by inducing inflammation, tissue destruction and proinflammatory cytokines production (Tzartos et al., 2008). This can contribute to increase MS activity.

Although several studies claim that SCFA may have an antiinflamatory effect, a controversy exists. It has been described that butyrate suppresses demyelination and enhances remyelination in mice with cuprizone-induced demyelination (Chen et al., 2019) and oral propionate can suppress the potential deleterious activities of T helper cells while Treg frequencies in all treated individuals increased significantly (Haghikia et al., 2016). However, as we have mentioned above, previous metabolomic studies performed in MS patients have also shown higher levels of acetate in the serum of MS patients in comparison with healthy controls (Moussallieh et al., 2014). Furthermore, it has been recently published (Qiu et al., 2019) that ex vivo acetate treatment increases IFN-*γ* production by exhausted T cells; thus, hyporesponsive T cells can be epigenetically remodeled and reactivated by acetate. Therefore, acetate, although it is a SCFA like propionate and butyrate, does not seem to share with them their beneficial effects.

Furthermore, other studies support a dual effect for them: depending on its levels and the immunological context of the host, SCFA could modulate T cells differentiation into both regulatory and effector cells (Park et al., 2015; Park et al., 2016; Mizuno et al., 2017). Recently, it has been published that SCFAs can induce CD4+ effector T cells, which are highly inflammatory, when transferred into mice. The authors suggest that in contrast to the moderate protective effect of SCFA, mice deficient in their receptors are more resistant to EAE pathogenesis. Thus, SCFA and their receptors could have the potential to regulate autoimmune CNS inflammation both positively and negatively (Park et al., 2019).

One limitation of the study could be the lack of information about the diet of patients, since diet can modify the composition of the microbiota. However, our aim was to describe, for the first time, plasma SCFA levels in MS patients and to study a possible relationship of these levels with immune cells, regardless of the cause or origin of SCFA levels. In future studies, we will take dietary data into account in order to go further investigating the role of SCFA and microbiota in MS. Another limitation is the absence of SCFA measurements in the CSF; it would be very interesting to know if SCFA levels in serum correlate with those in CSF to be able to understand the role that these SCFA could have in the interaction / modulation of the CNS cells.

## Conclusions

To sum up, our study shows that there are increased acetate levels in the plasma of MS patients; furthermore, they correlate with higher EDSS and MSSS. Higher values of acetate, propionate and butyrate also correlate with augmented CD8+ IL-17+ T cells, but only in MS patients. These results show that inflammatory background should be taken into account when exploring the association of microbiota metabolites and the immune system, and those basal patient conditions may change the influence of microbiota in the immune cells. Finally, our results strongly suggest that these bacterial metabolites can be part of the mechanisms by which microbiota may influence MS pathology. Future studies will completely elucidate the role of these molecules in this disease.

##  Supplemental Information

10.7717/peerj.10220/supp-1 Supplemental Information 1 Set of data used for analysis Data used for the analyses performed and reported in this article.

10.7717/peerj.10220/supp-2 Supplemental Information 2 LC/MS original data 1 LC-MS raw results with the quantification of acetate, propionate and butyrate (part 1).

10.7717/peerj.10220/supp-3 Supplemental Information 3 LC/MS original data 2 LC-MS raw results with the quantification of acetate, propionate and butyrate (part 2).

10.7717/peerj.10220/supp-4 Supplemental Information 4 Example of the gating strategy A gate including lymphocytes and monocytes and excluding debris and apoptotic cells was first established.

10.7717/peerj.10220/supp-5 Supplemental Information 5 Representative flow cytometry plots of a MS patient showing an inverse correlation between acetate levels with naïve CD4+ T lymphocytes and a direct correlation with CD8+ T cells producing IL-17

10.7717/peerj.10220/supp-6 Supplemental Information 6 Original results of the Pilot Study

10.7717/peerj.10220/supp-7 Supplemental Information 7 Demographic and clinical data and acetate concentration

## Additional Information and Declarations

### Data Availability

The following information was supplied regarding data availability: The raw measurements are available in the Supplementary Files. Gating strategy is available as Fig. S1. Representative flow cytometry plots are available as Fig. S2.
