# Supplementary material for: Acetate correlates with disability and immune response in multiple sclerosis
Source: PeerJ. 2020 Nov 16;8:e10220. doi: 10.7717/peerj.10220 (PMC7676361; doi:10.7717/peerj.10220)
Supplement: Supplemental Information 2 — LC-MS raw results with the quantification of acetate, propionate and butyrate (part 1). [file peerj-08-10220-s002.pdf]

RESULTADOS

Identificación del Cliente	Técnico Responsable
**Usuario (IP): Roberto Álvarez Lafuente**<br>**Facultad de Óptica**<br>**Departamento: Bioquímica**<br>**Universidad Complutense de Madrid**	**Estefanía García/ Cristina Gutiérrez**<br><br>Fecha análisis: **26/09/17-29/09/17**
Número de solicitud de servicio: **17-495;17-496;17-501**<br>Fecha de recepción de muestra/s: **26/09/17-28/09/17**<br>Código/s de muestra/s: 2017/EM/2109-2017/EM/2173; 2017/EM/2181-2017/EM/2194<br>Referencia de los análisis: **Acetato, propionato, butirato**<br>Tipo de análisis: **Análisis Cuantitativo mediante MRM (LC-QQQ-MS)**	

Analytical Conditions

- Injection volume: 10 µL
- Gradient mode: 20% phase B for 2 min, to 40% phase B to 7 min; from 40 to 100 % phase B till 7.5 min; Return to initial conditions from 8-9 min
- Phase A: H2O + 0.01% FA
  Phase B: Acetonitrile + 0.01% FA
- Flow: 0.6 mL/min
- Run time: 10 min
- MRM Transitions

  Acetate
  Quantifier (m/z): 194.0 >152.1(CE:18 V)
  Qualifier (m/z): 194.0 >137.05 (CE: 21 V)

  Propionate
  Quantifier (m/z): 208.2 >137.05(CE:20 V)
  Qualifier (m/z): 208.2 >165.15 (CE: 15 V)

  Butyrate
  Quantifier (m/z): 222.1 >137.00 (CE:20 V)
  Qualifier (m/z): 222.1 >152.05 (CE: 16 V)

- Column: Phenomenex Gemini 5u C18 110 A 150x2mm

Standard preparation

The standard mix with 500 mg/L of acetic, propionic and butyric acid (sigma) was prepared in acetonitrile:water (1:1) solution. Derivatization was carried out mixing 40 µL of standard mix with 20 µL of 200 mM 3-NPH and 20 µL of 120 mM EDC in 6% pyridine. The incubation time was 30 min and 40 ºC. Then samples were dissolved in 1920 µL of 10% acetonitrile and diluted in 1:1 ratio with ACN:H_2O (1:1). Finally samples were filtered with 0.22 µm PTFE filters and analyze by LC-ESI-QQQ 8030 Shimadzu mass spectrometer.

3-NPH: 3-Nitrophenylhydrazine hydrochloride

EDC: N-(3-dimethylaminopropyl)-N'-ethylcarbodiimide hydrochloride

Plasma sample preparation

Proteins were precipitated with equal volume of ACN:H_2O (1:1). After 10 minutes of centrifugation, at 4 ºC, derivatization was carried out mixing 40 µL of samples with 20 µL of 3-NPH and 20 µL of 120 mM EDC in 6% pyridine. The incubation time was 30 min and 40 ºC. Then samples were diluted with 920 µL of 10% acetonitrile. Finally samples were filtered with 0.22 µm PTFE filters and analyze by LC-ESI-QQQ 8030 Shimadzu mass spectrometer.

Results

Sample name	Acetate Concentration (µmol/L)	Propionate Concentration (µmol/L)	Butyrate Concentration (µmol/L)
11440	27,9	4,2	5,1
11444	29,5	5,5	4,7
11597	30,6	ND	3,0
11604	24,7	ND	ND
11906	29,6	7,3	ND
11907	31,1	7,1	3,2
11918	22,8	6,3	4,4
11929	32,9	6,8	5,2
1	63,2	7,6	9,1
2	26,5	4,4	3,4
3	72,4	8,7	6,6
4	52,8	5,7	4,5
5	49,2	5,0	4,8
6	53,8	9,2	5,1
7	157,8	5,9	4,7
8	49,1	3,7	4,5
9	49,4	5,6	6,2
10	49,1	3,9	4,3
11	384,3	15,4	9,3
12	46,3	6,4	5,5
13	38,8	5,5	4,5
14	64,7	6,6	8,9
15	57,6	8,1	8,1
16	15,1	3,1	3,1
17	26,1	5,9	6,1
18	23,4	3,6	3,6
19	13,3	4,4	4,4
20	21,9	4,3	3,9
21	40,3	4,5	4,9
22	34,4	3,8	5,2
11714	25,0	6,1	ND
11715	22,7	6,0	ND
11728	18,1	6,3	3,7
11620	33,8	5,0	5,8
11591	24,4	4,1	4,2
23	23,6	3,3	2,9
24	27,1	3,5	4,6
25	25,0	5,2	5,1
26	19,1	2,3	4,2
27	46,7	4,7	5,1
28	38,4	4,8	ND
29	23,9	3,8	2,2
30	21,0	2,3	ND

31	17,2	2,5	3,0
32	22,4	2,8	3,2
33	23,9	2,7	3,5
34	27,9	3,6	4,1
35	34,9	3,3	4,0
36	30,8	3,5	5,3
37	51,0	3,3	5,2
38	30,3	4,9	3,0
39	31,3	3,4	4,5
40	38,3	3,7	3,2
41	32,7	2,7	2,5
42	30,3	3,7	4,3
43	24,4	3,5	3,0
44	16,7	2,8	3,2
45	17,0	2,9	3,2
46	21,0	2,8	3,5
47	32,5	4,1	4,4
11562	22,5	ND	3,4
11564	24,3	ND	ND
11579	22,6	ND	4,4
48	25,5	ND	1,6
49	11,7	ND	ND
50	24,3	ND	ND
51	15,0	2,1	ND
52	14,0	1,7	ND
53	15,8	1,8	2,7
54	18,4	1,9	2,7
55	19,6	1,9	2,0
56	14,0	2,0	2,1
57	25,9	2,3	3,4
58	8,2	2,4	2,1
