# Supplementary material for: Acetate correlates with disability and immune response in multiple sclerosis
Source: PeerJ. 2020 Nov 16;8:e10220. doi: 10.7717/peerj.10220 (PMC7676361; doi:10.7717/peerj.10220)
Supplement: Supplemental Information 3 — LC-MS raw results with the quantification of acetate, propionate and butyrate (part 2). [file peerj-08-10220-s003.pdf]

RESULTADOS

<table>
<tr><td>Identificación del Cliente</td><td>Técnico Responsable</td></tr>
<tr><td rowspan="2">Usuario (IP): Roberto Álvarez Lafuente
Facultad de Óptica
Departamento: Bioquímica
Universidad Complutense de Madrid</td><td>Estefanía García/ Cristina Gutiérrez</td></tr>
<tr><td>Fecha análisis: 25/05/17-09/06/17</td></tr>
<tr><td colspan="2">Número de solicitud de servicio: 17-334;17-369;17-370;17-377
Fecha de recepción de muestra/s: 25/05/17; 05/06/17-09/06/17
Código/s de muestra/s: 2017/EM/1399-2017/EM/1407; 2017/EM/1526-2017/EM/1587; 2017/EM/1599-2017/EM/1628
Referencia de los análisis: Acetato, propionato, butirato
Tipo de análisis: Análisis Cuantitativo mediante MRM (LC-QQQ-MS)</td></tr>
</table>

Analytical Conditions

- Injection volume: 10 µL
- Gradient mode: 20% phase B for 2 min, to 40% phase B to 7 min; from 40 to 100 % phase B till 7.5 min; Return to initial conditions from 8-9 min
- Phase A: H_2O + 0.01% FA
  Phase B: Acetonitrile + 0.01% FA
- Flow: 0.6 mL/min
- Run time: 10 min
- MRM Transitions

  Acetate
  Quantifier (m/z): 194.0 >152.1(CE:18 V)
  Qualifier (m/z): 194.0 >137.05 (CE: 21 V)

  Propionate
  Quantifier (m/z): 208.2 >137.05(CE:20 V)
  Qualifier (m/z): 208.2 >165.15 (CE: 15 V)

  Butyrate
  Quantifier (m/z): 222.1 >137.00 (CE:20 V)
  Qualifier (m/z): 222.1 >152.05 (CE: 16 V)

- Column: Phenomenex Gemini 5u C18 110 A 150x2mm

Standard preparation

The standard mix with 500 mg/L of acetic, propionic and butyric acid (sigma) was prepared in acetonitrile:water (1:1) solution. Derivatization was carried out mixing 40 µL of standard mix with 20 µL of 200 mM 3-NPH and 20 µL of 120 mM EDC in 6% pyridine. The incubation time was 30 min and 40 ºC. Then samples were dissolved in 1920 µL of 10% acetonitrile and diluted in 1:1 ratio with ACN:H_2O (1:1). Finally samples were filtered with 0.22 µm PTFE filters and analyze by LC-ESI-QQQ 8030 Shimadzu mass spectrometer.

3-NPH: 3-Nitrophenylhydrazine hydrochloride

EDC: N-(3-dimethylaminopropyl)-N'-ethylcarbodiimide hydrochloride

Plasma sample preparation

Proteins were precipitated with equal volume of ACN:H_2O (1:1). After 10 minutes of centrifugation, at 4 ºC, derivatization was carried out mixing 40 µL of samples with 20 µL of 3-NPH and 20 µL of 120 mM EDC in 6% pyridine. The incubation time was 30 min and 40 ºC. Then samples were diluted with 920 µL of 10% acetonitrile. Finally samples were filtered with 0.22 µm PTFE filters and analyze by LC-ESI-QQQ 8030 Shimadzu mass spectrometer.

Results

Chromatogram of standard mix 50 µg/L

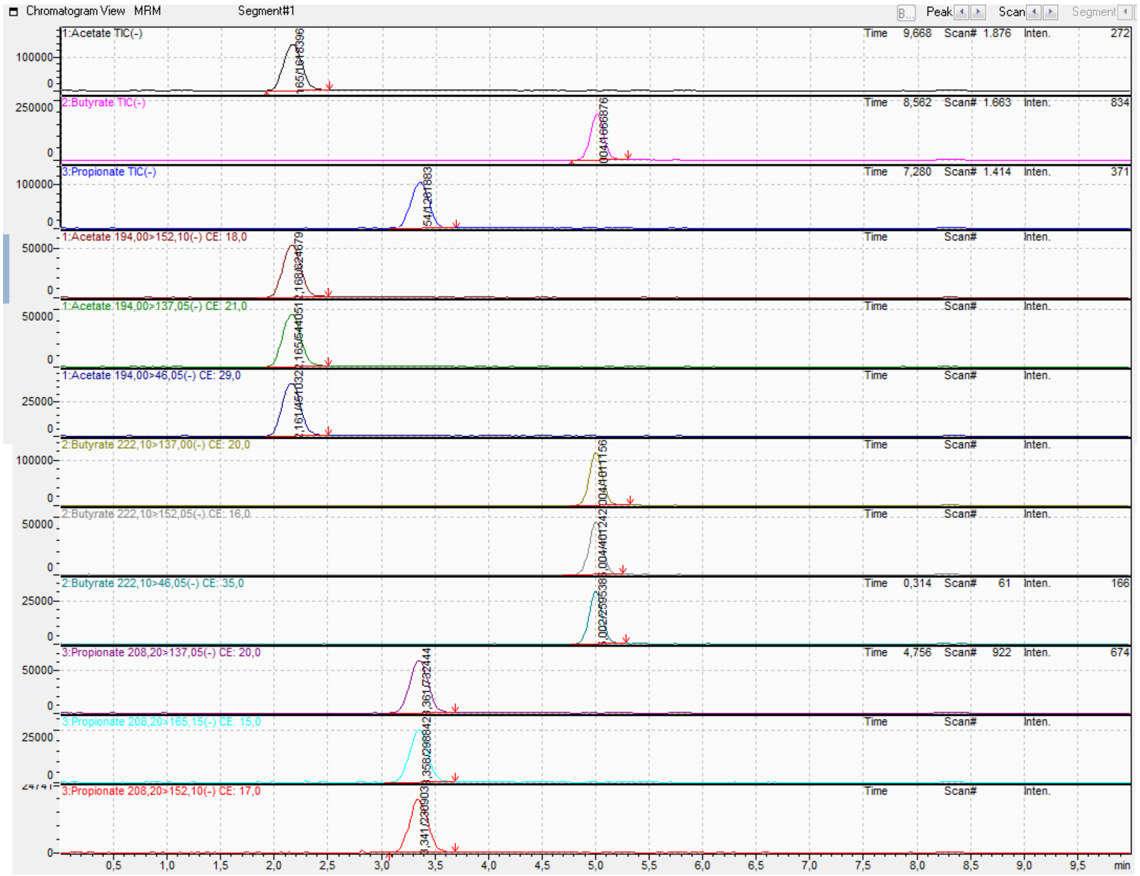

Calibration curve

Acetate

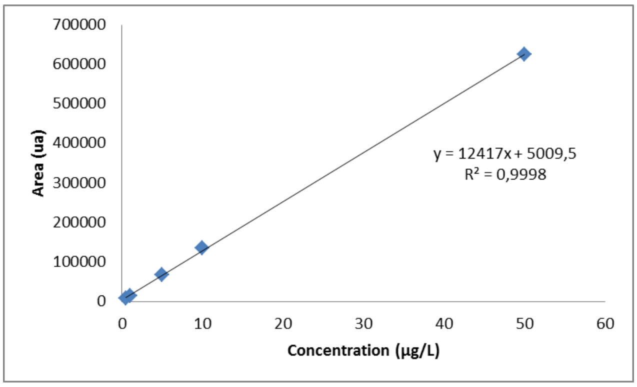

LD (detection limit) = 0.99 µg/L

LQ (quantification limit) = 3.29 µg/L

Propionate

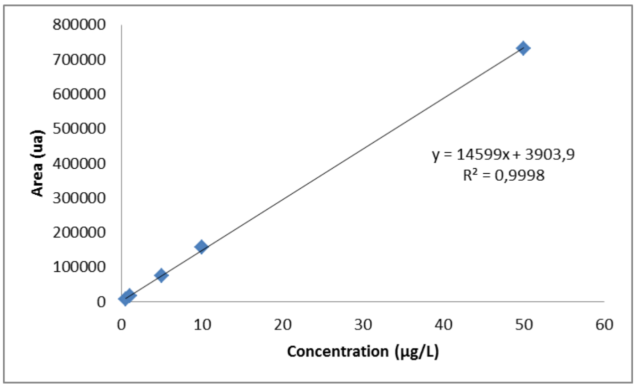

LD (detection limit) = 0.67 µg/L

LQ (quantification limit) = 2.24 µg/L

Butyrate

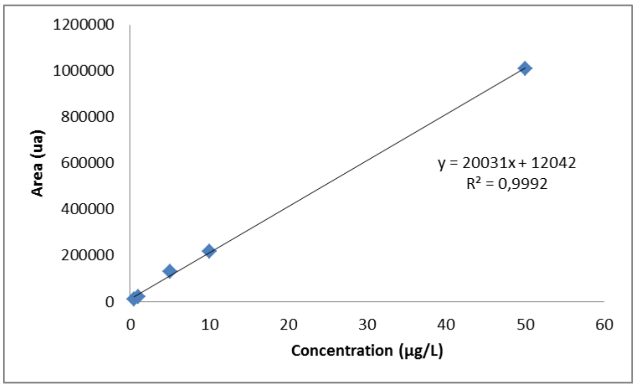

LD (detection limit) = 0.56 µg/L

LQ (quantification limit) = 1.86 µg/L

Chromatogram sample 11719

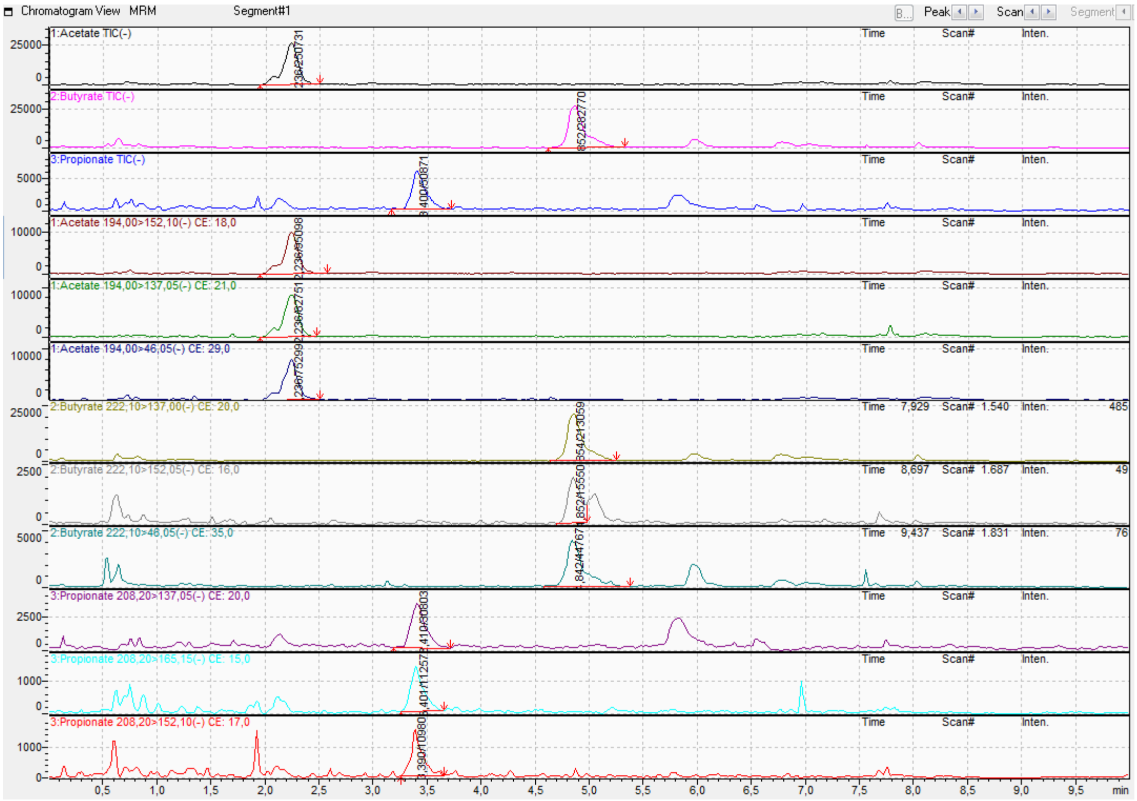

Sample name	Acetate Concentration (μmol/L)	Propionate Concentration (μmol/L)	Butyrate Concentration (μmol/L)
11121	9,8	4,3	2,3
11720	51,8	2,9	2,3
11719	18,7	4,4	2,4
11690	15,6	3,9	1,8
11726	12,8	2,9	3,8
11697	14,2	3,1	1,7
11700	18,2	3,8	1,5
11687	11,7	3,2	2,3
11689	13,5	3,0	1,6
9379	19,6	3,0	1,8
9790	17,1	2,8	0,0
9094	21,5	1,3	1,4
8814	13,3	2,4	2,3
9636	16,6	2,4	1,7
9771	15,2	3,3	2,0
9585	25,0	2,4	2,5
9043	15,8	2,7	0,4
9471	12,4	2,6	1,6
8684	25,2	1,5	0,5
9391	19,2	4,9	1,7
9801	12,7	2,6	0,9

9386	35,2	3,9	2,4
9401	15,4	3,4	8,9
8896	14,9	4,8	4,2
9609	23,0	3,6	1,0
11701	16,5	4,6	5,0
11702	36,7	7,5	6,1
11703	16,0	2,0	0,0
11711	19,5	3,7	5,9
11716	22,2	4,4	3,8
11728	13,0	3,7	4,1
11729	20,5	3,5	4,3
11730	10,2	4,0	5,2
11731	14,4	2,7	7,2
9195	14,5	3,3	4,8
9457	26,1	3,0	8,2
9614	15,7	2,0	3,6
9772	55,5	9,1	1,9
10011	66,2	9,1	0,0
10121	51,2	7,5	1,7
10178	26,8	1,6	1,6
10179	31,0	1,9	0,0
8911	45,5	3,1	2,4
8982	22,2	2,6	0,0
9051	36,0	3,1	1,5
9059	24,0	2,2	0,0
9118	46,0	1,7	0,0
9660	27,2	3,6	0,0
9991	38,9	1,7	0,0
10519	29,0	ND	0,0
11732	53,5	6,0	0,0
11734	29,6	5,7	0,0
11735	29,6	9,0	0,0
11738	19,1	8,5	4,6
11741	13,0	6,0	4,3
11743	15,8	7,8	6,2
11744	23,5	12,1	5,6
9676	31,5	10,6	5,6
10351	23,3	10,1	5,5
10416	19,3	5,0	3,7
10456	19,8	9,1	5,2
10546	21,2	7,9	5,8
10594	17,5	10,6	5,1
10952	33,3	14,9	7,3
11174	22,1	9,1	7,5
11183	19,7	7,1	4,5
9777	32,2	10,1	4,4
10738	29,1	13,0	7,5
1745	32,3	12,3	3,7
10968	34,7	11,2	4,8

11158	33,8	12,6	6,3
11173	22,3	8,1	5,8
11285	18,5	11,8	5,9
10859	22,7	11,8	6,8
11239	44,1	7,8	4,9
