# Supplementary figures and images for: Acetate correlates with disability and immune response in multiple sclerosis
Source: PeerJ. 2020 Nov 16;8:e10220. doi: 10.7717/peerj.10220 (PMC7676361; doi:10.7717/peerj.10220)

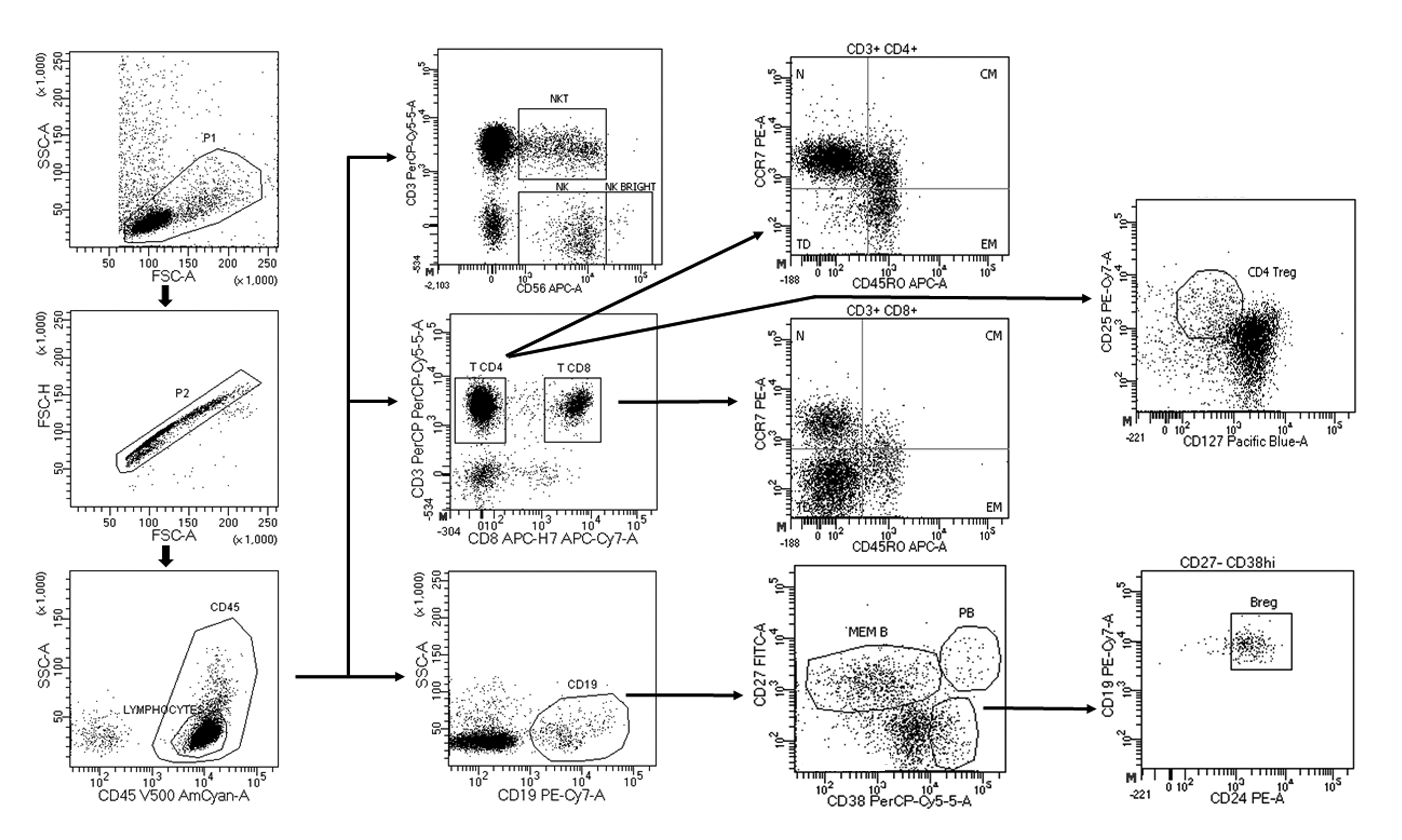

Supplement: Supplemental Information 4 — A gate including lymphocytes and monocytes and excluding debris and apoptotic cells was first established. [file peerj-08-10220-s004.jpg]

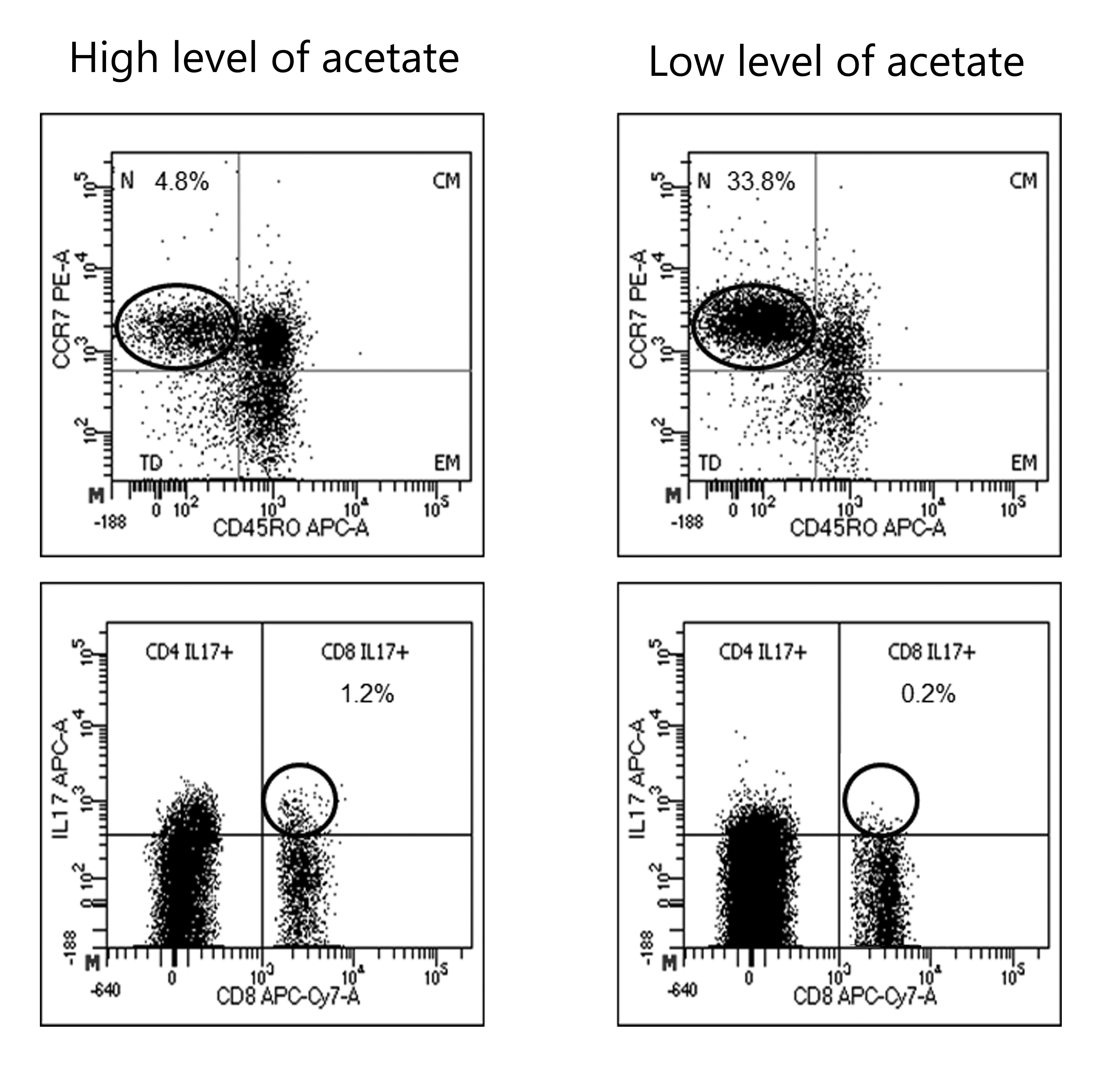

Supplement: Supplemental Information 5 [file peerj-08-10220-s005.jpg]
