# Supplementary material for: Acetate correlates with disability and immune response in multiple sclerosis
Source: PeerJ. 2020 Nov 16;8:e10220. doi: 10.7717/peerj.10220 (PMC7676361; doi:10.7717/peerj.10220)
Supplement: Supplemental Information 6 [file peerj-08-10220-s006.pdf]

Results

Sample name	Acetate Concentration (µM)	Propionate Concentration (µM)	Butyrate Concentration (µM)
1	3,4	0,0	0,2
2	5,0	0,0	0,3
3	5,8	0,1	0,6
4	11,7	0,0	0,5
5	24,9	0,0	9,9
6	51,9	0,5	10,8
7	16,6	0,0	3,7
8	21,6	0,0	5,5
9	24,5	0,0	8,0
10	41,1	1,5	13,8
11	32,6	1,3	43,9
12	32,8	6,0	66,2
13	35,3	14,5	15,7
14	29,9	3,8	25,5
15	33,0	6,1	33,1
16	39,7	4,3	10,3
17	27,9	3,2	14,2
18	53,5	4,3	16,8
19	16,2	2,6	9,4
20	42,8	2,9	12,5
