# Supplementary material for: Acetate correlates with disability and immune response in multiple sclerosis
Source: PeerJ. 2020 Nov 16;8:e10220. doi: 10.7717/peerj.10220 (PMC7676361; doi:10.7717/peerj.10220)
Supplement: Supplemental Information 7 [file peerj-08-10220-s007.docx]

**Supplementary Table 1.** Demographic and clinical data and acetate concentration.

| **Sample name** | **Gender** | **Age**  **(years)** | **EDSS** | **Acetate Concentration (μM)** |
| --- | --- | --- | --- | --- |
| 1 | M | 38 | 0.0 | 3.4 |
| 2 | F | 38 | 2.0 | 5.0 |
| 3 | F | 34 | 0.0 | 5.8 |
| 4 | F | 25 | 1.0 | 11.7 |
| 5 | M | 44 | 0.0 | 24.9 |
| 6 | M | 32 | 3.5 | 51.9 |
| 7 | F | 60 | 0.0 | 16.6 |
| 8 | M | 41 | 2.5 | 21.6 |
| 9 | F | 43 | 1.0 | 24.5 |
| 10 | F | 30 | 6.0 | 41.1 |
| 11 | F | 38 | 2.5 | 32.6 |
| 12 | F | 37 | 5.0 | 32.8 |
| 13 | M | 38 | 4.5 | 35.3 |
| 14 | M | 35 | 4.0 | 29.9 |
| 15 | F | 39 | 5.5 | 33.0 |
| 16 | F | 40 | 3.5 | 39.7 |
| 17 | F | 51 | 0.0 | 27.9 |
| 18 | M | 36 | 5.5 | 53.5 |
| 19 | F | 18 | 0.0 | 16.2 |
| 20 | F | 46 | 6.0 | 42.8 |

|  |  |  |  |
| --- | --- | --- | --- |
|  |  |  |  |
